# Effects of Initial Combinations of Gemigliptin Plus Metformin Compared with Glimepiride Plus Metformin on Gut Microbiota and Glucose Regulation in Obese Patients with Type 2 Diabetes: The INTESTINE Study

**DOI:** 10.3390/nu15010248

**Published:** 2023-01-03

**Authors:** Soo Lim, Minji Sohn, Jose C. Florez, Michael A. Nauck, Jiyoung Ahn

**Affiliations:** 1 Department of Internal Medicine, Seoul National University Bundang Hospital, Seoul National University College of Medicine, Seongnam 13620, Republic of Korea; 2 Diabetes Unit and Center for Genomic Medicine, Massachusetts General Hospital, Harvard Medical School, Boston, MA 02114, USA; 3 Diabetes Division, Katholisches Klinikum Bochum, St. Josef Hospital (Ruhr University Bochum), 44791 Bochum, Germany; 4 Department of Population Health, New York University Grossman School of Medicine, New York, NY 10016, USA

**Keywords:** antidiabetic drugs, type 2 diabetes, intestinal flora, dipeptidyl peptidase-4 inhibitor, sulfonylurea

## Abstract

The efficacy and safety of medications can be affected by alterations in gut microbiota in human beings. Among antidiabetic medications, incretin-based therapy such as dipeptidyl peptidase 4 inhibitors might affect gut microbiomes, which are related to glucose metabolism. This was a randomized, controlled, active-competitor study that aimed to compare the effects of combinations of gemigliptin–metformin vs. glimepiride–metformin as initial therapies on gut microbiota and glucose homeostasis in drug-naïve patients with type 2 diabetes. Seventy drug-naïve patients with type 2 diabetes (mean age, 52.2 years) with a glycated hemoglobin (HbA1c) level ≥7.5% were assigned to either gemigliptin–metformin or glimepiride–metformin combination therapies for 24 weeks. Changes in gut microbiota, biomarkers linked to glucose regulation, body composition, and amino acid blood levels were investigated. Although both treatments decreased the HbA1c levels significantly, the gemigliptin–metformin group achieved HbA1c ≤ 7.0% without hypoglycemia or weight gain more effectively than did the glimepiride–metformin group (59% vs. 24%; *p* < 0.05). At the phylum level, the Firmicutes/Bacteroidetes ratio tended to decrease after gemigliptin–metformin therapy (*p* = 0.065), with a notable depletion of taxa belonging to Firmicutes, including *Lactobacillus*, *Ruminococcus torques*, and *Streptococcus* (all *p* < 0.05). However, regardless of the treatment modality, a distinct difference in the overall gut microbiome composition was noted between patients who reached the HbA1c target goal and those who did not (*p* < 0.001). Treatment with gemigliptin–metformin resulted in a higher achievement of the glycemic target without hypoglycemia or weight gain, better than with glimepiride–metformin; these improvements might be related to beneficial changes in gut microbiota.

## 1. Introduction

Globally, fewer than 30% of patients with diabetes reach glycated hemoglobin (HbA1c) levels of <7.0% (53 mmol/mol), indicating that there is an unmet medical need for more optimal glycemic diabetes management [1]. Several studies have shown that early combination therapy is effective in achieving glycemic target goals [2]. Moreover, intensive glucose control in the early period after diagnosis has been shown to reduce the risk of micro- and macrovascular complications and mortality, even in the long term [3].

Because of their good glucose-lowering efficacy, sulfonylureas have been widely used together with metformin for rapid glucose control in patients with type 2 diabetes [4]. However, the possibility of increased risk of hypoglycemia and likely weight gain associated with this therapy, combined with the availability of newer agents with fewer side effects and additional benefits of preventing cardiovascular events, has led to a reduction in the frequency of prescription of sulfonylureas [5].

Dipeptidyl peptidase 4 (DPP-4) inhibitors are oral anti-hyperglycemic agents with proven glucose-lowering efficacy that have been developed relatively recently [6]. This incretin-based therapy has several advantages, including for example a low risk of hypoglycemia and weight neutrality [7]. Based on these advantages, a DPP-4 inhibitor is preferred over sulfonylurea therapy, particularly for patients at risk of hypoglycemia [6]. Several phase 3 studies and registry studies on patients with type 2 diabetes have demonstrated that the addition of a DPP-4 inhibitor to metformin is non-inferior in terms of glucose-lowering efficacy compared with the addition of sulfonylurea [8].

Dysbiosis of the gut microbiota is now recognized as a major contributor to chronic human diseases, including type 2 diabetes [9]. A recent animal model study reported that DPP-4 inhibitor therapy promoted a functional shift in the gut microbiome, contributing to improved glucose regulation [10]. Thus, changes in the intestinal microflora have emerged as contributors to the action of antidiabetic agents [11]. Furthermore, the gut microbiota plays significant roles in the metabolism and disease status of the host during antidiabetic treatment, indicating that it might be a novel therapeutic target [12].

Gemigliptin is a relatively new DPP-4 inhibitor with proven efficacy and safety in various clinical situations [13,14]. It has high selectivity for DPP-4, resulting in substantial increases in the levels of intact, biologically active glucagon-like peptide-1 and glucose-independent insulinotropic polypeptide [15]. In the present study, we investigated the effect of combined treatment with gemigliptin–metformin on glucose regulation, the gut microbiota, and biomarkers related to glucose metabolism. Changes in the levels of serum amino acids and biomarkers linked to inflammation were also compared with those obtained for the combined treatment with glimepiride–metformin in drug-naïve patients with type 2 diabetes.

## 2. Materials and Methods

### 2.1. Study Design and Participants

This study was a proof-of-concept, active drug-controlled, randomized controlled trial (RCT) performed at the Seoul National University Bundang Hospital (SNUBH, Seongnam, Republic of Korea) from 2017 to 2021. Individuals with type 2 diabetes and obesity were eligible if they were aged ≥20 years, had not received any antidiabetic agents during the previous 6 weeks, and had a body mass index (BMI) ≥25 kg/m^2^ at the screening visit. Participants were excluded if they had type 1 diabetes, were pregnant or lactating, had New York Heart Association class III or IV heart failure, had undergone gastrointestinal surgery, or showed substantially decreased kidney function (serum creatinine [Cr] levels ≥1.5 mg/dL for men and ≥1.4 mg/dL for women).

A total of 70 participants were assigned randomly (1:1) to either gemigliptin 50 mg with metformin 1000 mg/day (gemigliptin–metformin group) or glimepiride 2 mg with metformin 1000 mg/day (glimepiride–metformin group; Supplementary Figure S1 and Figure S2). The random sequence was generated using a statistical program, and the allocation was kept hidden from the physician who recruited the subjects. All participants were educated by the study coordinators to keep a healthy lifestyle during the study period.

### 2.2. Study Endpoints

The primary endpoint was the change in the gut microbiota (Firmicutes/Bacteroidetes ratio) after 24 weeks of treatment. Detailed analyses were conducted at the genus and species levels. The secondary endpoint was the change in HbA1c levels from the baseline to the 24-week time point. The exploratory endpoints were changes in the levels of circulatory amino acids—surrogate markers for pancreatic β-cell function and insulin resistance—and body composition. For safety assessment, adverse events including hypoglycemia were assessed during the study period. Hypoglycemic episodes were determined based on participants’ symptoms reflecting hypoglycemia and a self-monitored plasma glucose level <70 mg/dL.

### 2.3. Measurements

The BMI was calculated by dividing the subject’s weight (kg) by their height squared (m^2^). Clinical parameters including blood pressure and body weight were measured using standard methods. Blood pressure was measured with the subjects in a seated position using an electronic blood pressure meter (UA-1020 device; A&D, Tokyo, Japan). Blood pressure was measured twice 5 min apart, and the mean value was used in the analysis.

HbA1c levels were measured at SNUBH, a National Glycohemoglobin Standardization Program Level II-certified laboratory, using the Bio-Rad Variant II Turbo Hemoglobin Testing System (Bio-Rad Laboratories, Hercules, CA, USA) on a high-performance liquid chromatography analyzer. Fasting plasma and postprandial 2 h glucose concentrations (FPG and PP2, respectively) were analyzed using the hexokinase method. Fasting plasma insulin levels were measured by radioimmunoassay (Linco, St. Louis, MO, USA). The plasma concentration of C-peptide was measured by radioimmunoassay (Izotop, HoilBioMed, Seoul, Republic of Korea). Triglyceride levels were measured using the glycerol-3-phosphate oxidase peroxide method, and high-density lipoprotein and low-density lipoprotein cholesterol levels were measured by relevant enzymatic assays. Aspartate and alanine aminotransferase (AST and ALT, respectively) levels were measured using the reduced nicotinamide adenine dinucleotide ultraviolet method, and serum creatinine (Cr) was measured by Jaffe’s kinetic method using a Hitachi 747 chemistry analyzer (Hitachi, Tokyo, Japan).

All subjects underwent a standardized 75 g oral glucose tolerance test (OGTT) with overnight fasting for 10 h at the baseline and after 6 months. The levels of plasma glucose, insulin, and C-peptide were measured at the baseline and at 30 min, 60 min, and 2 h after the OGTT. The area under the OGTT curve of glucose concentration (AUCglucose) was derived using trapezoidal integration. The homeostasis models of assessment of insulin resistance (HOMA-IR) and pancreatic β-cell function (HOMA-β) were calculated as: (glucose (mg/dL)) × insulin (mg/dL)/405) and (360 × insulin (μIU/mL))/(glucose (mg/dL) − 63), respectively.

Serum high-sensitivity C-reactive protein (hsCRP) was measured using a high-sensitivity automated immunoturbidimetric method (Roche, Basel, Switzerland). Plasminogen activator inhibitor-1 (PAI-1) level was measured using an ELISA kit (RayBiotech, Peachtree Corners, GA, USA).

Amino acid levels were calculated using the Cliquid Software (SCIEX, Framingham, MA, USA) from the plasma peak area ratio analyzed by liquid chromatography–tandem mass spectrometry using the aTRAQ reagent (SCIEX, Framingham, MA, USA) with internal standards. Branched-chain amino acids (BCAAs) were defined as the sum of leucine, isoleucine, and valine, whereas aromatic amino acids (AAAs) were defined as the sum of phenylalanine, tryptophan, and tyrosine.

While the subjects were in a fasting state, body composition was assessed using multifrequency bioelectrical impedance analytical machines (Inbody720, InBody, Seoul, Republic of Korea), followed by validation by dual-energy X-ray absorptiometry or computed tomography [16,17]. The participants were requested to refrain from smoking, drinking alcohol, and strenuous exercise for 48 h before the measurements.

### 2.4. Stool Collection and 16s rRNA Amplicon Sequencing

Fecal samples were collected and frozen within 3 days of the visit date in a sterile kit provided by the research team. Fecal bacterial genomic DNA extraction was performed using Mag-Bind^®^ Universal Pathogen kits (Omega Bio-Tek, Norcross, GA, USA). The fecal sample was suspended in 275 μL of SLX-Mlus buffer, followed by bead pulverization in a mixer mill MM400 (Retsch, Haan, Germany) with further isolation, cleaning, and elution procedures being carried out according to the manufacturer’s protocols. The preparation of ribosomal RNA gene amplicon samples for the Illumina MiSeq System (Illumina, San Diego, CA, USA) was achieved using a method for preparing samples for sequencing the variable V3–V4 regions of the 16S rRNA gene. The extracted fecal microbial DNA was amplified with the 16S Amplicon PCR Forward Primer (5′–TCGTCGGCAGCGTCAGATGTGTATAAGAGACAGCCTACGGGNGGCWGCAG–3′) and the 16S Amplicon PCR Reverse Primer (5′–GTCTCGTGGGCTCGGAGAT GTGTATAAGAGACAGGACTACHVGGGTATCTAATCC–3′). These amplicon primers, 2× KAPA HiFi HotStart ReadyMix (Roche, Basel, Switzerland), and DNA were used in PCR under conditions of 3 min at 95 °C, followed by 25 cycles at 95 °C for 30 s, annealing at 55 °C for 30 s, and extension at 72 °C for 30 s, and a final extension at 72 °C for 5 min. Subsequently, sample DNAs were cleaned with HiAccuBead kits (AccuGene, Deerfield Beach, FL, USA) and a magnetic stand. Index PCR was performed using the IDT indexing primer (Integrated DNA Technologies, Coralville, IA, USA) for the Illumina MiSeq System, 2× KAPA HiFi HotStart ReadyMix, and PCR-grade water. Polymerase chain reaction was carried out at 95 °C for 3 min; followed by 8 cycles of 95 °C for 30 s, 55 °C for 30 s, and 72 °C for 30 s, then 72 °C for 5 min and holding at 4 °C. After the cleanup step, the concentrations of DNA libraries were verified using Qubit 4.0 with 1× dsDNA HS assay solution (Thermo Fisher Scientific, Waltham, MA, USA) and sequenced using the Illumina MiSeq system. Reads were sorted using unique barcodes for each PCR product. The barcode, linker, and primer sequences were then removed from the original sequencing reads. The sequencing results were analyzed, and the taxonomic assignment was performed using the Silva RNA reference database (https://www.arb-silva.de/ accessed on 29 April 2021).

### 2.5. Statistical Analysis

The number of study participants was calculated based on 80% power (at α = 0.05), to conservatively detect a 25% difference in the Firmicutes/Bacteroidetes ratio between the two groups, which was based on previous studies showing increased Bacteroidetes and decreased Firmicutes in subjects with type 2 diabetes after DPP-4 inhibitor treatment [18,19]. Descriptive statistics were used for the baseline characteristics, which are summarized as means and standard deviations (SDs) or medians and interquartile ranges. The analysis was performed according to the intention-to-treat principle, including all available measurements with multiple imputations.

For microbiota, rarified counts were used for analysis including α-diversity (richness and Shannon index) (GUniFrac and vegan R packages). Taxa were selected for analysis if they were present in at least 25% of the samples and had a mean relative abundance greater than 0.01%, to exclude unnecessary comparisons. The gut microbial overall structure between groups was visualized by principal coordinates analysis (PCoA) and permutational multivariate analysis of variance (PERMANOVA) using Bray–Curtis dissimilarities at the genus level for (i) the intention-to-treat population; (ii) those who reached the HbA1c target of ≤7.0% (53 mmol/mol); and (iii) those who did or did not gain body weight. To identify clinically relevant genera, a linear discrimination analysis (LDA) with adjustment for visit date (http://huttenhower.sph.harvard.edu/galaxy/ accessed on 2 September 2021) and an analysis of the composition of microbiomes (ANCOM) with adjustment for age, sex, visit date, and baseline values were performed (ANCOMBC R package). Following the distribution, paired *t*-tests or Wilcoxon signed-rank tests were applied to detect differences in the gut microbial features at the baseline and post-treatment measurements in each treatment arm. Pearson’s correlation coefficient was tested between clinical parameters and changes in microbiota log counts and amino acid levels. A Phylogenetic Investigation of Communities by Reconstruction of Unobserved States (PICRUSt) analysis (the metagenomics R package) was used to predict metagenome function against the Greengenes database [20]. *p*-values were adjusted using the Benjamini–Hochberg method for multiple comparisons of microbiota and amino acids. All statistical analyses were performed using the R software version 4.0.2 (R Development Core Team, Vienna, Austria) and RStudio version 1.3.1056 (RStudio, Boston, MA, USA).

### 2.6. Study Approval

Our study was carried out in accordance with the Declaration of Helsinki (2013) and in compliance with the ethical principles of the International Council on Harmonisation Good Clinical Practice Guidelines. An independent ethics committee approved the study protocol (B-1507-308-008). This trial was registered at ClinicalTrials.gov (NCT02609815). All participants provided written informed consent before being screened for eligibility.

## 3. Results

### 3.1. Baseline Characteristics and Their Changes in the Study Participants

The baseline characteristics of the intention-to-treat study population are shown in Table 1. Most parameters were largely well balanced between the gemigliptin–metformin and the glimepiride–metformin groups, with the exception of systolic blood pressure (SBP). Both groups had HbA1c levels ≥8.0% (64 mmol/mol) at the baseline; moreover, about 40% of the participants had hypertension and 70% had dyslipidemia.

### 3.2. Changes in Clinical Profiles

After 24 weeks of treatment, body weight, BMI, and waist circumference increased significantly by the glimepiride–metformin combination therapy but these did not alter by the gemigliptin-metformin combination therapy, resulting in no significant difference between the groups (Table 2). The gemigliptin–metformin combination therapy decreased whole-body fat percentage significantly. The abdominal visceral fat area (VFA) was also decreased by the gemigliptin-metformin combination therapy, but it did not achieve statistical significance (Table 2).

**Figure 1:**
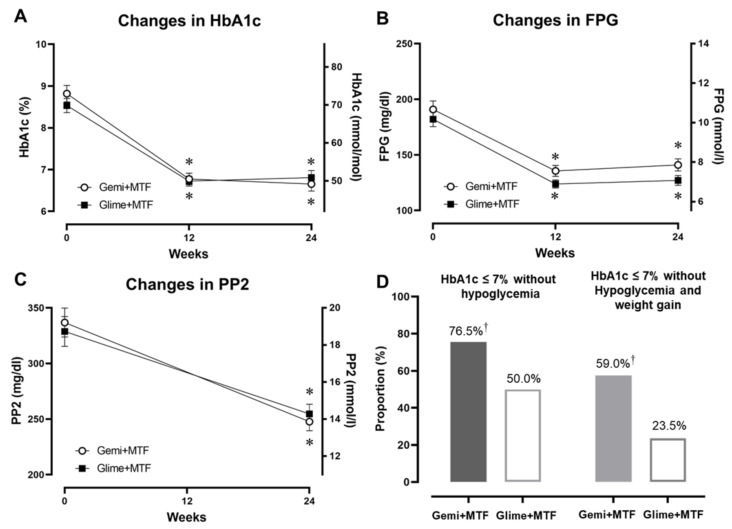
Changes in glycemic parameters after gemigliptin–metformin or glimepiride–metformin combination therapies during the study period, and the proportion of participants who achieved the glycemic target of HbA1c ≤7.0% (53 mmol/mol) without hypoglycemia or weight gain. (**A**) HbA1c (%), (**B**) FPG, fasting plasma glucose concentration, (**C**) PP2 (postprandial 2-h glucose concentration), and (**D**) proportion of participants within the glycemic target without hypoglycemia (left) and without hypoglycemia and weight gain (right). * *p* < 0.05 from the baseline, † *p* < 0.05 between the groups. Key: Gemi, gemigliptin; Glime, glimepiride; MTF, metformin.

Both groups showed a significant decrease in HbA1c levels; however, there was a slightly greater non-significant decrease in the gemigliptin–metformin group than in the glimepiride–metformin group (−2.1% vs. −1.7%; *p* = 0.082; Table 2 and Figure 1A). The fasting glucose concentrations decreased significantly in both groups (Figure 1B,C). At the end of the study, the proportion of participants with HbA1c ≤7.0% (53 mmol/mol) and without hypoglycemia was significantly higher in the gemigliptin–metformin combination group compared with the glimepiride–metformin combination group (77% vs. 50%, *p* < 0.05) (Figure 1D). Similarly, the proportion of participants who achieved this glycemic target goal without hypoglycemia and weight gain was also higher in the gemigliptin–metformin combination group than in the glimepiride–metformin combination group (59.0% vs. 23.5%, *p* < 0.05).

The gemigliptin–metformin combination treatment decreased the proinsulin/insulin ratio significantly, whereas the glimepiride–metformin combination treatment did not, resulting in a significant difference between the groups (*p* < 0.05) (Table 2). Both groups exhibited a significant decrease in the HOMA-IR. In the assessment of inflammatory markers, the gemigliptin–metformin combination treatment decreased PAI-1 and hsCRP levels significantly, whereas the glimepiride–metformin combination treatment only decreased the PAI-1 level slightly (Table 2).

### 3.3. Changes in Gut Microbiota Profiles

**Figure 2:**
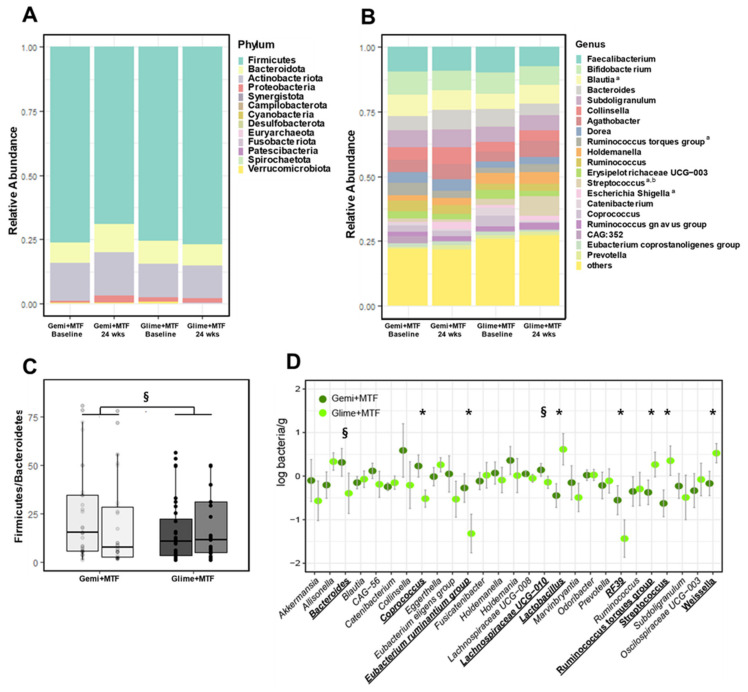
Changes in the microbiota profiles of bacterial phyla with gemigliptin–metformin or glimepiride–metformin combination therapies at the baseline and at the end of the study. (**A**) compositional profiling of bacterial phyla. (**B**) Compositional profiling of the top 20 bacterial genera. (**C**) Firmicutes/Bacteroidetes ratio. (**D**) Comparison of the logarithmic mean changes in bacterial abundance from the baseline to the 24-week time point between treatment groups. ^a^ *p* < 0.1 in changes after gemigliptin–metformin therapy; ^b^ *p* < 0.1 in changes after glimepiride–metformin therapy. * *p* < 0.05, ^§^ *p* < 0.1. Key: Gemi, gemigliptin; Glime, glimepiride; MTF, metformin.

The overall microbial α-diversity and β-diversity were not different between the two treatment groups (Supplementary Figure S3A,B). However, Firmicutes, which was the predominant phylum accounting for over 70% of the gut microbiota, decreased significantly after gemigliptin-based therapy (Figure 2A,B). *Proteobacteria* tended to increase with gemigliptin-based therapy. The Firmicutes/Bacteroidetes ratio, as a marker of metabolic derangement, decreased in the gemigliptin group, resulting in a between-group difference with tendency to significance (*p* = 0.065) (Figure 2C). Consistent with this, we noticed a pronounced depletion of multiple genera and species belonging to Firmicutes (*Lactobacillus*, *Ruminococcus torques*, *Streptococcus*, and *Weissella*, all *p* < 0.05; Figure 2D).

### 3.4. Differences in Gut Microbiota Associated with Clinical Outcomes and Predicted Functional Pathways

After the intervention, regardless of the treatment modality, a distinct difference in overall gut microbiome composition was noted between the participants who reached the HbA1c target goal and those who did not (PERMANOVA; *p* < 0.001; Figure S4A,B). This altered overall structure was particularly characterized by enriched *Eubacterium eligens*, *Odoribacter*, *Holdemania*, and *Lachnospiraceae*, and by depleted *Collinsella*, *Blautia*, and *Subdoligranulum*. There were borderline differences between the participants who gained weight and those who did not (PERMANOVA, *p* = 0.086). The identified genera also exhibited correlations with the changed values (Supplementary Figure S5).

The group difference for the changes in the functional composition estimated by PICRUSt after the 24-week treatment in the two groups is shown in Supplementary Figure S6. The microbial metabolisms, including biotin, glycerophospholipid, glycolysis/gluconeogenesis, and histidine metabolism, were decreased after gemigliptin–metformin therapy, whereas they were increased after the glimepiride–metformin therapy, resulting in a significant difference between the treatment groups.

### 3.5. Changes in Amino Acid Levels

In the gemigliptin–metformin group, alanine and glutamine levels were increased significantly (Supplementary Table S1). In the glimepiride–metformin group, AAA levels were increased significantly. Glycine levels, which are generally low in subjects with metabolic disorders, were increased in both groups.

For measures of positive glucose regulation, the α-amino-n-butyric acid level showed a positive correlation with the AUCglucose and was decreased in both groups (Supplementary Figure S7). The ethanolamine level, which was positively correlated with AUCglucose and body weight, and the tyrosine level, which was positively correlated with the proinsulin/insulin ratio, were decreased in the gemigliptin–metformin group and increased in the glimepiride–metformin group, leading to a significant difference at week 24 (*p* < 0.05). The arginine, glutamic acid, and leucine levels, which are reported to be associated with insulin resistance [21], were not significantly changed in either of the treatment groups. The *Bacteroides* genus alone was negatively correlated with histidine and tryptophan.

### 3.6. Adverse Events

In this study, three subjects in the gemigliptin group and eleven in the glimepiride group experienced adverse events (9% vs. 32%; *p* < 0.05) (Table 3). In particular, seven subjects in the glimepiride group experienced hypoglycemia, and two of them wanted to discontinue the study. There was no case of hypoglycemia in the gemigliptin–metformin group.

## 4. Discussion

In this RCT, the proportion of participants with HbA1c ≤ 7.0% (53 mmol/mol) but lacking hypoglycemia was significantly greater in the gemigliptin–metformin combination group compared with the glimepiride–metformin combination group. In addition, the proportion of participants who achieved this goal without hypoglycemia or weight gain was more than double in the gemigliptin–metformin combination group vs. the glimepiride–metformin combination group. This outcome suggests that an early combination of gemigliptin–metformin is preferable and safer for the treatment of drug-naïve patients with type 2 diabetes with moderately high HbA1c levels.

Of note, the initial combination treatment of gemigliptin–metformin increased the abundance of the gut microbiota associated with improvements in glucose regulation and reduced body fat. At the phylum level, the Firmicutes and Firmicutes/Bacteroidetes bacteria, which are markers that are well known to be linked to obesity [22], decreased with the gemigliptin–metformin therapy but not with the glimepiride–metformin therapy, leading to a significant between-group difference. Firmicutes abundance was positively correlated with abdominal visceral adiposity in our study. Firmicutes possess enzymes involved in lipid and carbohydrate metabolism, leading to the increased absorption of calories from ingested food and, subsequently, to fat accumulation [23].

In this study, a better glycemic control was associated with several genera of gut microbiota: with high abundance of the *Eubacterium eligens* group, *Odoribacter*, and *Holdemania* (Figure S4B). Both the *Eubacterium eligens* group and *Odoribacter* are reported to produce short-chain fatty acids, which help maintain normal intestinal function and increase insulin sensitivity [24,25]. By contrast, *Blautia*, which is known to be associated with glucose dysregulation [26], was enriched in patients who did not reach HbA1c <7%. In the correlation of genera and clinical values, the *Eubacterium eligens* group showed an inverse correlation with HOMA-IR.

Here, weight gain in the study participants was associated with *Streptococcus* and *Allisonella* (Figure S4B). By contrast, a lack of weight gain was associated with a high abundance of *Bacteroides*, *Oscillospiraceae* UCG-003, *Akkermansia*, the *Eubacterium ruminantium* group, and RF39. Members of the *Allisonella* genus were reported to be more abundant in Europeans with a high waist–hip ratio and inflammatory index [27]. *Akkermansia* is a well-known constituent of microbiota possessing an anti-obesity property [28]. In a cross-sectional study comprising 6896 Chinese participants, there was a dose-related association between an increase in the abundance of *Akkermansia* and a decrease in metabolic risk [29]. By contrast, in a large cohort study performed in the USA, *Streptococcus* was enriched and RF39 was depleted in subjects with obesity compared with participants with a healthy weight [30].

Intriguingly, the two therapies altered the gut microbiota in different ways in this study, which might contribute to better glucose control in the gemigliptin-based therapy. *Bacteroides* is the most abundant genus in the human gastrointestinal system, comprising >30% of all bacteria [31]. The abundance of *Bacteroides* tended to increase with the gemigliptin–metformin therapy, but not with the glimepiride–metformin therapy. Sitagliptin administration reversed the decrease in the abundance of *Bacteroides* induced by a high-fat diet in mice, followed by increased succinate levels and improved glucose tolerance and insulin sensitivity [10].

Conversely, the glimepiride–metformin therapy decreased *Eubacterium ruminantium* and RF39 and increased *Streptococcus*, leading to significant differences between the two groups (Figure 2D). In a cohort of 531 Finnish men with metabolic syndrome, RF39 was associated with a low BMI [32], consistent with our results, which revealed negative correlations between RF39 abundance and BMI and fat composition. *Streptococcus* is one of the most abundant genera in the gastrointestinal tract, and it is increased in metabolically unhealthy conditions, such as diabetes mellitus and obesity [33].

In the estimated microbial functional analysis, gemigliptin-based therapy altered several metabolic pathways (Supplementary Figure S6). Among them, the decrease in glycolysis/gluconeogenesis afforded by gemigliptin-based therapy might contribute to a better glycemic control compared with glimepiride–metformin therapy. Imidazole propionate, which is a histidine metabolite, was reported as being increased in patients with type 2 diabetes [34]. This might be relevant to the decrease in the abundance of *Streptococcus*, one of the imidazole propionate-producing microbiota. In addition, biotin biosynthesis was increased in individuals with impaired glucose regulation, indicating the microbial response to a lack of biotin in the disease status [35].

Moreover, several studies have shown that metformin changes the gut microbiota favorably such as increasing the abundance of *Akkermansia*, thus rendering them more similar to the microbiota of a healthy host [36,37]. When metformin-treated human feces were transferred to germ-free mice, the glucose regulation was improved [38], in addition to changes in microbiota abundance, which could be related to improving the intestinal barrier integrity and regulating the bile acid metabolism [39]. As changes in the microbiota can further affect other bacteria with indirect environmental shifts and bacterium–bacterium interactions [38], the initial combination with metformin in our study might have attenuated the alteration in the gut microbiota that is supposedly induced by gemigliptin or glimepiride therapy. However, the aim of our study was to investigate the changes of gut microbiota with combination therapy, which is more frequently prescribed than metformin alone.

In the amino acid analysis, serum methionine levels were decreased after both treatments. A high methionine level is associated with hyperhomocysteinemia and insulin resistance [40]. Methionine might also serve as a potential clinical biomarker of oxidative stress [41]. Remarkably, ethanolamine levels were decreased after gemigliptin-based therapy, whereas they were increased significantly after glimepiride-based therapy. Ethanolamine is utilized as a source by diverse bacteria; specifically, Firmicutes contain various unique genes involved in ethanolamine utilization [42].

Increased AAA levels, particularly tyrosine, are associated with insulin resistance and obesity [43]. A previous RCT found that metformin decreased the concentrations of tyrosine and phenylalanine, indicating that reducing the levels of AAAs could improve glucose metabolism [44]. In our study, gemigliptin–metformin therapy and glimepiride–metformin changed the tyrosine levels differently, with significant group differences (*p* < 0.05), indicating that this change was caused by mechanisms beyond metformin itself.

In this study, the proinsulin/insulin ratio decreased significantly after gemigliptin–based therapy, but not after glimepiride–metformin therapy. The change from proinsulin to insulin is a critical process in pancreatic β-cells for glucose regulation [45]. This increased ratio is used effectively as a marker of islet cell distress or compromised insulin secretion [46]. In addition, the gemigliptin–metformin combination treatment decreased the hsCRP and PAI-1 levels significantly. Both molecules are well-known inflammatory markers related to the vascular complications of type 2 diabetes [47]. Taken together, the favorable changes in gut microbiota by gemigliptin–metformin therapy might be implicated in the control of dysbiosis-related inflammation [48].

Therapy with DPP-4 inhibitors improves glycemic control with a similar efficacy as that of sulfonylureas; however, they do not induce hypoglycemia or weight gain [7]. Moreover, large prospective trials of such drugs have demonstrated their cardiovascular safety [7]. Based on this, DPP-4 inhibitors are now the preferred choice over sulfonylureas for the majority of patients on metformin treatment [49]. Patients with type 2 diabetes treated with the initial combination of vildagliptin plus metformin showed effectively improved glucose levels with a significantly greater reduction in glycemic variability and hypoglycemia than did patients treated with metformin [6].

Gemigliptin has several advantages over other glucose-lowering agents. It was more effective than glimepiride in reducing glycemic variability as an initial combination therapy with metformin in patients with type 2 diabetes [14]. Compared with dapagliflozin, gemigliptin has proven beneficial effects on glucose variability assessed by continuous glucose monitoring [14]. These findings are likely to be associated with high glycemic target goal achievement without hypoglycemia.

In this RCT of drug-naïve Korean patients with type 2 diabetes, 77% of the participants who received the gemigliptin–metformin combination therapy achieved the glycemic target goal of HbA1c ≤ 7.0% (53 mmol/mol) without hypoglycemia. This approach altered the gut microbiota and amino acid levels favorably, suggesting potential improvements in pancreatic β-cell function and anti-inflammatory effects. Our findings suggest that changes in the gut microbiota play important roles in augmenting the efficacy of gemigliptin therapy. This may apply to DPP-4 inhibitors in more general terms.

## 5. Conclusions

In this RCT of drug-naïve Korean patients with type 2 diabetes, 77% of the patients treated with the gemigliptin–metformin combination therapy achieved the glycemic target goal of HbA1c ≤ 7.0% (53 mmol/mol) without hypoglycemia. This approach favorably altered the gut microbiota and amino acid levels, suggesting potential improvements in pancreatic β-cell function and anti-inflammatory effects. Our findings suggest that changes in the gut microbiota are important for augmenting the efficacy of gemigliptin therapy. This may apply to DPP-4 inhibitors in more general terms.

## Figures and Tables

**Table 1:** Baseline characteristics of the intention-to-treat study population.

	Gemigliptin + Metformin (*n* = 34)	Glimepiride + Metformin (*n* = 34)	Total	*p*
Age (years)	50.9 ± 12.0	53.6 ± 9.5	52.2 ± 10.8	0.291
Male sex	25 (74%)	23 (68%)	48 (71%)	0.594
Body weight (kg)	77.7 ± 11.8	77.4 ± 11.3	77.5 ± 11.5	0.892
BMI (kg/m^2^)	28.1 ± 3.0	27.8 ± 3.1	28.0 ± 3.0	0.598
Waist circumference, cm	94.4 ± 8.6	93.3 ± 9.1	93.9 ± 8.8	0.621
SBP, mm Hg	137.3 ± 16.1	130.1 ± 11.7	133.7 ± 14.5	0.041
DBP, mm Hg	84.1 ± 12.5	80.1 ± 8.5	82.1 ± 10.8	0.126
Heart rate, beats per min	78.9 ± 12.2	79.8 ± 12.4	79.4 ± 12.2	0.768
HbA1c, %	8.8 ± 1.2	8.5 ± 1.0	8.7 ± 1.1	0.279
HbA1c, mmol/mol	72.9 ± 12.7	69.8 ± 10.8	71.3 ± 11.8	0.279
Fasting glucose, mg/dL	190.8 ± 44.7	182.1 ± 38.6	186.4 ± 41.7	0.390
Total cholesterol, mg/dL	212.6 ± 51.0	190.6 ± 37.8	201.4 ± 45.8	0.050
Triglyceride, mg/dL	220.8 ± 129.9	166.0 ± 116.5	193.0 ± 125.4	0.074
HDL-cholesterol, mg/dL	47.4 ± 8.7	47.4 ± 12.2	47.4 ± 10.6	0.996
LDL-cholesterol, mg/dL	126.8 ± 32.4	110.6 ± 27.3	118.6 ± 30.8	0.030
AST, IU/L	35.4 ± 14.6	38.9 ± 18.0	37.1 ± 16.3	0.394
ALT, IU/L	44.4 ± 18.7	47.5 ± 22.7	46.0 ± 20.7	0.543
eGFR, mL/min/1.73 m^2^	99.8 ± 15.7	96.8 ± 12.3	98.3 ± 14.1	0.389
Comorbidities				
Hypertension	13 (38%)	14 (41%)	27 (40%)	0.804
Dyslipidemia	25 (74%)	23 (68%)	48 (71%)	0.594
Medication use				
ACE inhibitor or ARB	6 (18%)	10 (29%)	16 (24%)	0.253
Lipid-lowering agents	8 (24%)	12 (35%)	20 (29%)	0.287

Data are the mean ± SD or n (%). Key: SBP, systolic blood pressure; DBP, diastolic blood pressure; eGFR, estimated glomerular filtration rate; ACE, angiotensin converting enzyme; ARB, angiotensin receptor blocker.

**Table 2:** Investigational parameters at the baseline and after 24 weeks of treatment.

Variables	Gemigliptin + Metformin (*n* = 34)			Glimepiride + Metformin (*n* = 34)			*p* for Delta
	Baseline	24 weeks	*p*	Baseline	24 weeks	*p*	
Body weight, kg	77.7 ± 11.8	78.3 ± 12.7	0.258	77.4 ± 11.3	78.9 ± 12.1	0.031	0.229
BMI, kg/m^2^	28.1 ± 3.0	28.3 ± 3.2	0.290	27.8 ± 3.1	28.5 ± 3.3	0.016	0.101
Waist circumference, cm	94.4 ± 8.6	94.5 ± 8.3	0.888	93.3 ± 9.1	95.5 ± 9.4	0.030	0.078
SBP, mm Hg	137.3 ± 16.1	134.5 ± 14.0	0.306	130.1 ± 11.7	131.3 ± 14.5	0.640	0.283
DBP, mm Hg	84.1 ± 12.5	82.2 ± 9.5	0.338	80.1 ± 8.5	77.9 ± 10.3	0.194	0.901
AST, IU/L	35.4 ± 14.6	34.2 ± 17.6	0.725	38.9 ± 18.0	39.9 ± 27.1	0.798	0.671
ALT, IU/L	44.4 ± 18.7	43.9 ± 34.9	0.917	47.5 ± 22.7	49.3 ± 36.0	0.726	0.750
eGFR, mL/min per 1.73 m^2^	99.8 ± 15.7	100.6 ± 17.0	0.615	96.8 ± 12.3	97.8 ± 12.9	0.286	0.886
Glucose homeostasis							
HbA1c, %	8.8 ± 1.2	6.7 ± 1.0	<0.001	8.5 ± 1.0	6.8 ± 1.0	<0.001	0.082
HbA1c, mmol/mol	72.9 ± 12.7	49.2 ± 10.7	<0.001	69.8 ± 10.8	50.9 ± 10.7	<0.001	0.082
Fasting glucose, mg/dL	190.8 ± 44.7	140.9 ± 31.5	<0.001	182.1 ± 38.6	127.1 ± 25.9	<0.001	0.603
C-peptide, mg/L	2.7 ± 0.9	2.6 ± 0.7	0.260	2.8 ± 0.9	2.8 ± 1.0	0.691	0.756
Insulin, μIU/mL	13.6 ± 8.2	13.1 ± 8.9	0.378	14.3 ± 7.2	16.3 ± 10.8	0.279	0.174
Proinsulin, pmol/L	8.5 ± 5.1	5.7 ± 2.9	<0.001	7.6 ± 3.7	8.9 ± 12.1	0.413	0.018
Proinsulin/Insulin	0.65 ± 0.23	0.46 ± 0.15	<0.001	0.63 ± 0.38	0.56 ± 0.37	0.797	0.015
HOMA-IR	6.4 ± 4.5	4.7 ± 3.7	<0.001	6.4 ± 3.7	5.3 ± 4.0	0.019	0.296
Oral glucose tolerance test							
Glucose at 0 min, mg/dL	185.5 ± 50.6	140.9 ± 28.7	<0.001	179.8 ± 44.5	127.5 ± 20.0	<0.001	0.474
Glucose at 30 min, mg/dL	273.4 ± 61.8	218.8 ± 33.5	<0.001	271.3 ± 52.4	219.4 ± 36.3	<0.001	0.835
Glucose at 60 min, mg/dL	348.0 ± 67.4	268.8 ± 40.8	<0.001	336.2 ± 62.7	278.7 ± 37.2	<0.001	0.168
Glucose at 120 min, mg/dL	336.8 ± 76.0	244.7 ± 47.8	<0.001	328.9 ± 78.6	254.7 ± 51.1	<0.001	0.274
AUCglucose, mg × h/dL	612.5 ± 121.4	463.8 ± 6	<0.001	597.2 ± 115.8	477.9 ± 63.7	<0.001	0.346
Inflammation							
PAI-1, mg/L	256.4 ± 49.0	223.7 ± 49.5	0.001	254.1 ± 34.2	236.5 ± 40.7	0.011	0.177
hsCRP, mg/L *	2.1 ± 4.1	1.0 ± 1.4	0.020	1.8 ± 2.3	1.9 ± 2.7	0.189	0.615
Body composition							
Muscle mass, kg	49.0 ± 9.4	50.0 ± 9.4	<0.001	49.7 ± 8.0	50.8 ± 8.3	0.006	0.899
Fat mass, kg	26.8 ± 8.3	25.4 ± 6.5	0.198	25.7 ± 7.1	25.8 ± 7.7	0.883	0.210
Fat percent, %	33.3± 6.7	32.2 ± 6.5	0.017	32.8 ± 6.2	32.2 ± 6.5	0.069	0.319
Abdominal VFA, cm^2^	121.7 ± 37.0	117.5 ± 36.6	0.068	117.5 ± 35.9	116.1 ± 38.4	0.435	0.332

* Values compared after logarithmic transformation. Key: AUC, area under the curve; HOMA-IR, homeostatic model assessment for insulin resistance; PAI, plasminogen activator inhibitor; hsCRP, high-sensitivity c-reactive protein; VFA, visceral fat area.

**Table 3:** Adverse events in both groups observed during the 24 weeks of treatment.

	Gemigliptin + Metformin (*n* = 34)	Glimepiride + Metformin (*n* = 34)
Any adverse event	3 (9%)	11 (32%)
Adverse event leading to study drug discontinuation	0	2 (6%) *
Any serious adverse event	0	0
Adverse event of special interest		
Hypoglycemia	0	7 (21%)
Hypoglycemia with symptom	0	6 (18%)
Hypoglycemia without symptom	0	1 (3%)
Tremor	0	1 (3%)
Dizziness	0	1 (3%)
Abdominal pain	1 (3%)	0
Abdominal distension	0	1 (3%)
Heartburn	0	1 (3%)
Constipation	1 (3%)	0
Asteatosis	1 (3%)	0
Varicose vein	0	1 (3%)

Data are *n* (%). * This study drug was discontinued because the patient developed hypoglycemia. No case of severe hypoglycemia was reported.

## Data Availability

Not applicable.

## Supplementary Materials

The following supporting information can be downloaded at: https://www.mdpi.com/article/10.3390/nu15010248/s1. Figure S1: Study design and structures of the intervention; Figure S2: Trial outline; Figure S3: Changes in microbiota profiles of bacterial genera with gemigliptin–metformin or glimepiride–metformin combination therapies during the study period; Figure S4: Profiles of gut microbiota related to clinical outcomes according to the treatment group; Figure S5: Heatmap of the Pearson’s correlation analysis between clinical parameters and the microbiota; Figure S6: Predicted functional composition of metagenomes based on the 16S rRNA gene sequencing data of the gemigliptin–metformin and glimepiride–metformin treatment. Heatmap of Kyoto Encyclopedia of Genes and Genomes (KEGG) pathways identified as changes of pathways with between-group differences; Figure S7: Heatmap of the Pearson’s correlation analysis between clinical parameters with changes in amino acids; Table S1: Changes in amino acids at the baseline and after 24 weeks of treatment with the gemigliptin–metformin or glimepiride–metformin combination.
