# Supplementary material for: Effects of Initial Combinations of Gemigliptin Plus Metformin Compared with Glimepiride Plus Metformin on Gut Microbiota and Glucose Regulation in Obese Patients with Type 2 Diabetes: The INTESTINE Study
Source: Nutrients. 2023 Jan 3;15(1):248. doi: 10.3390/nu15010248 (PMC9824054; doi:10.3390/nu15010248)
Supplement: Supplementary file 1 [file nutrients-15-00248-s001.zip › nutrients-2006877-supplementary.pdf]

Supplementary Materials

Supplementary Figure S1. Study design and structures of the intervention.

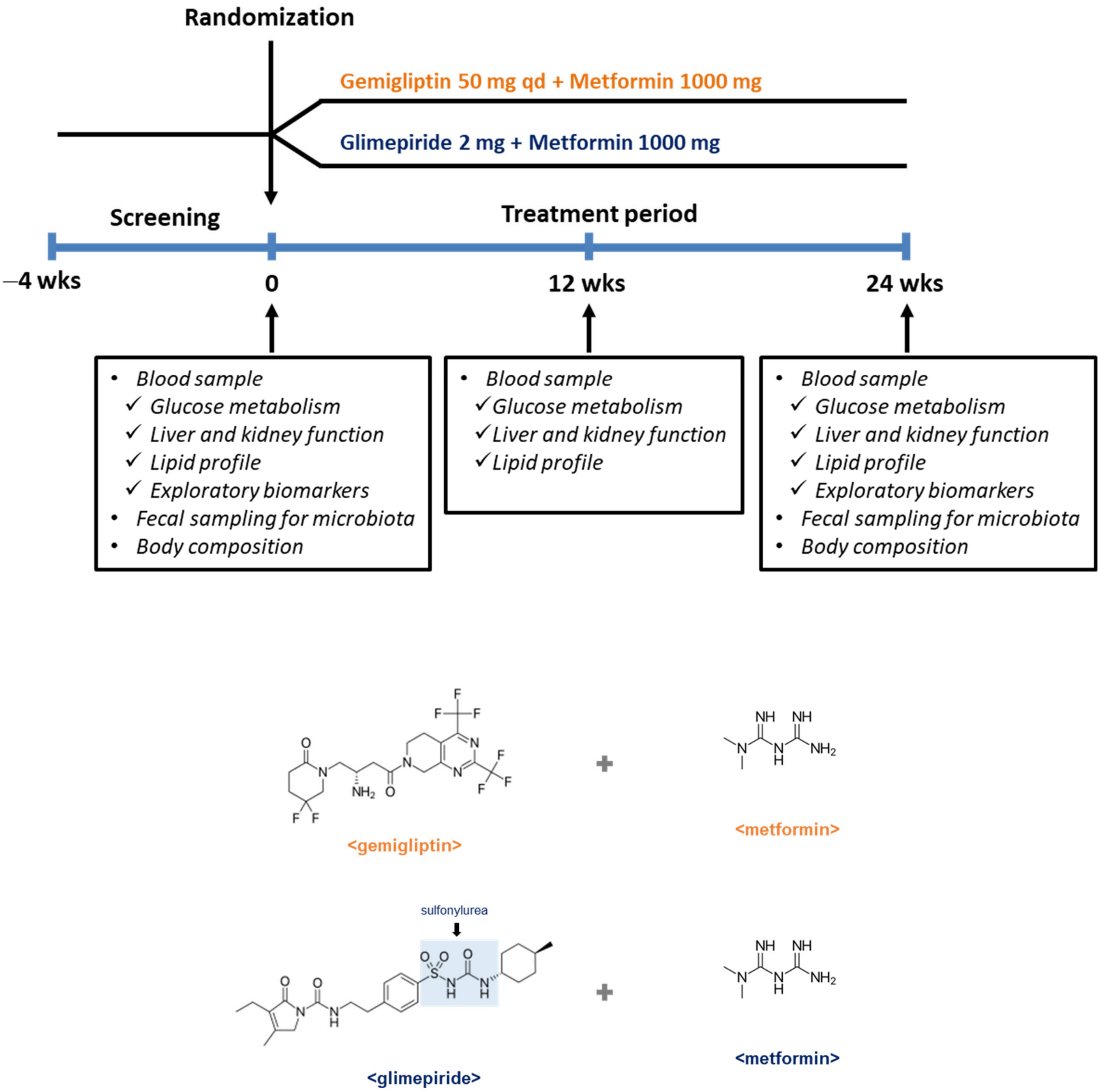

Supplementary Figure S2. Trial outline.

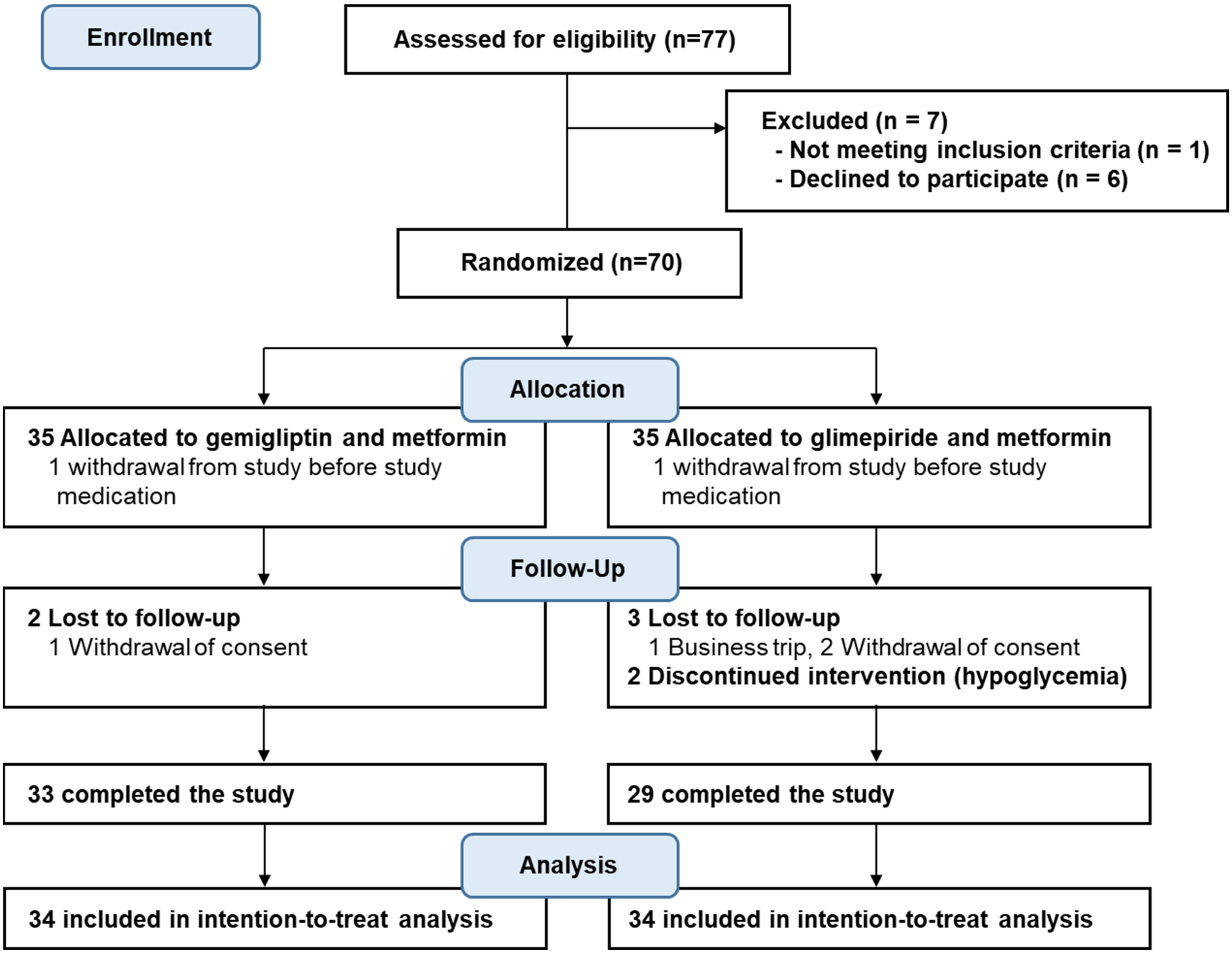

Supplementary Figure S3. Changes in microbiota profiles of bacterial genera with gemigliptin–metformin or glimepiride–metformin combination therapies during the study period. A: Community diversity by Shannon index. B: Dissimilarities in gut microbiota composition by PCoA. Key: Gemi, gemigliptin; Glime, glimepiride; MTF, metformin; PCoA, principal coordinates analysis

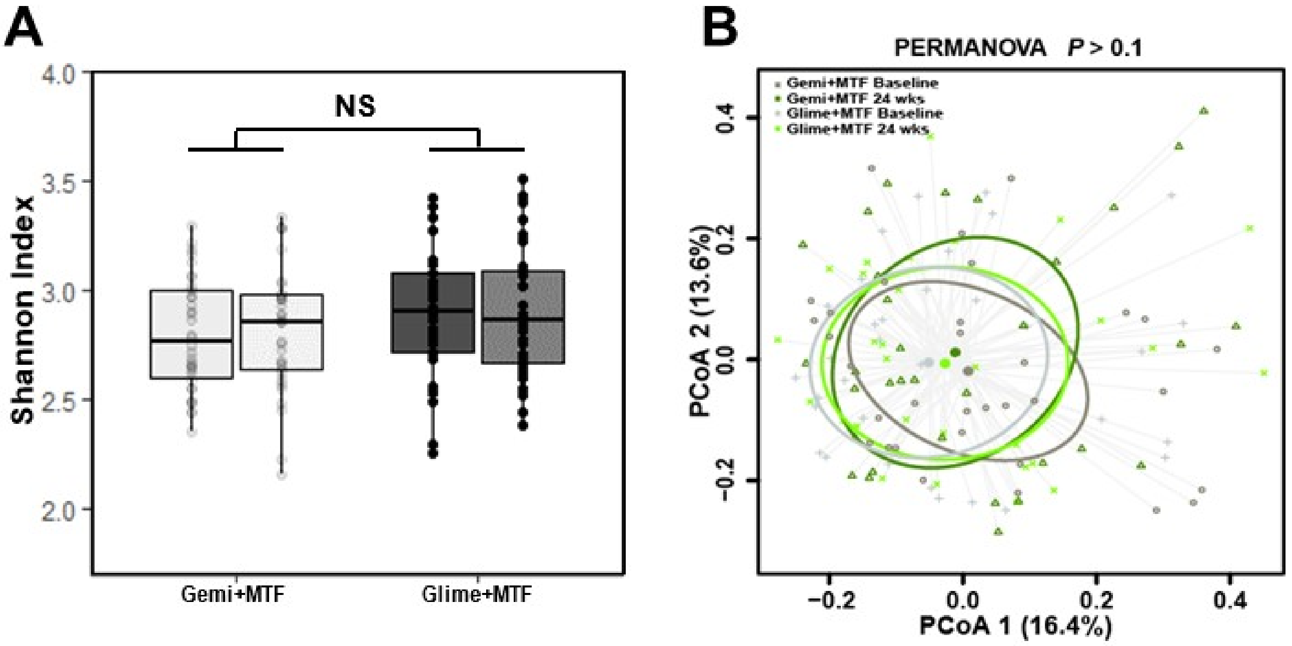

Supplementary Figure S4. Profiles of gut microbiota related to clinical outcomes according to the treatment group. A: Dissimilarities in gut microbiota composition by PCoA with (i) those who did or did not reach the target HbA1c level and (ii) those who did or did not gain weight. B: Gut microbial taxonomic biomarkers identified by the LDA or ANCOM with (i) those who did or did not reach the target HbA1c level and (ii) those who did or did not gain weight.

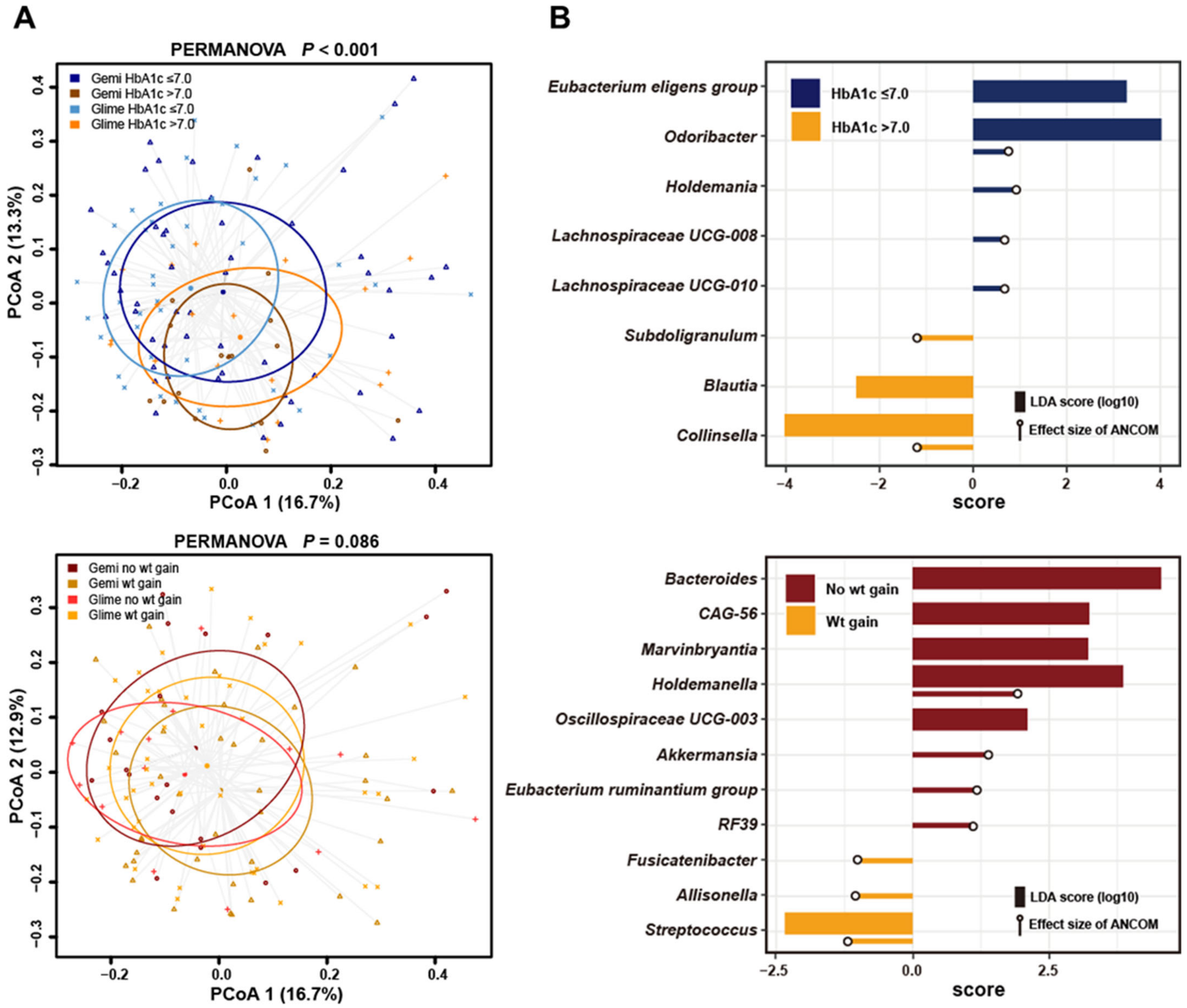

Supplementary Figure S5. Heatmap of the Pearson's correlation analysis between clinical parameters and the microbiota. *FDR-adjusted $p < 0.1$. Key: AUCglc, area under the curve of the oral glucose tolerance test; proins/ins, proinsulin/insulin ratio; HOMA-IR, homeostasis model assessment for insulin resistance; Bwt, body weight; WC, waist circumference; FM, fat mass; VFA, visceral fat area; PAI, plasminogen activator inhibitor; hsCRP, high-sensitivity C-reactive protein

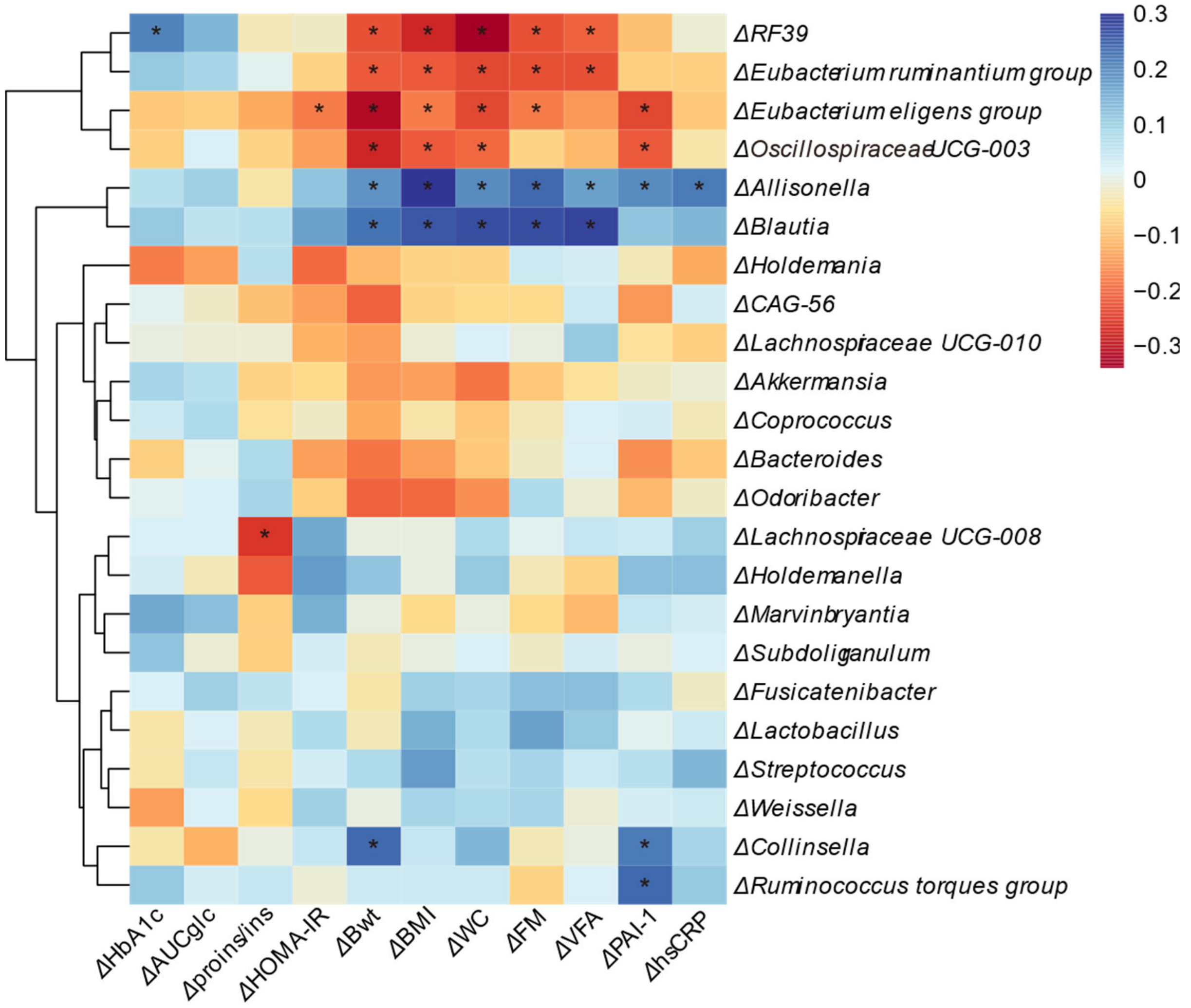

Supplementary Figure S6. Predicted functional composition of metagenomes based on the 16S rRNA gene sequencing data of the gemigliptin–metformin and glimepiride–metformin treatment. Heatmap of Kyoto Encyclopedia of Genes and Genomes (KEGG) pathways identified as changes of pathways with between-group differences. The values of color in the heatmap represent the changes of the normalized abundance of KEGG pathways before and after the two treatments. Key: Gemi, gemigliptin; Glime, glimepiride; MTF, metformin

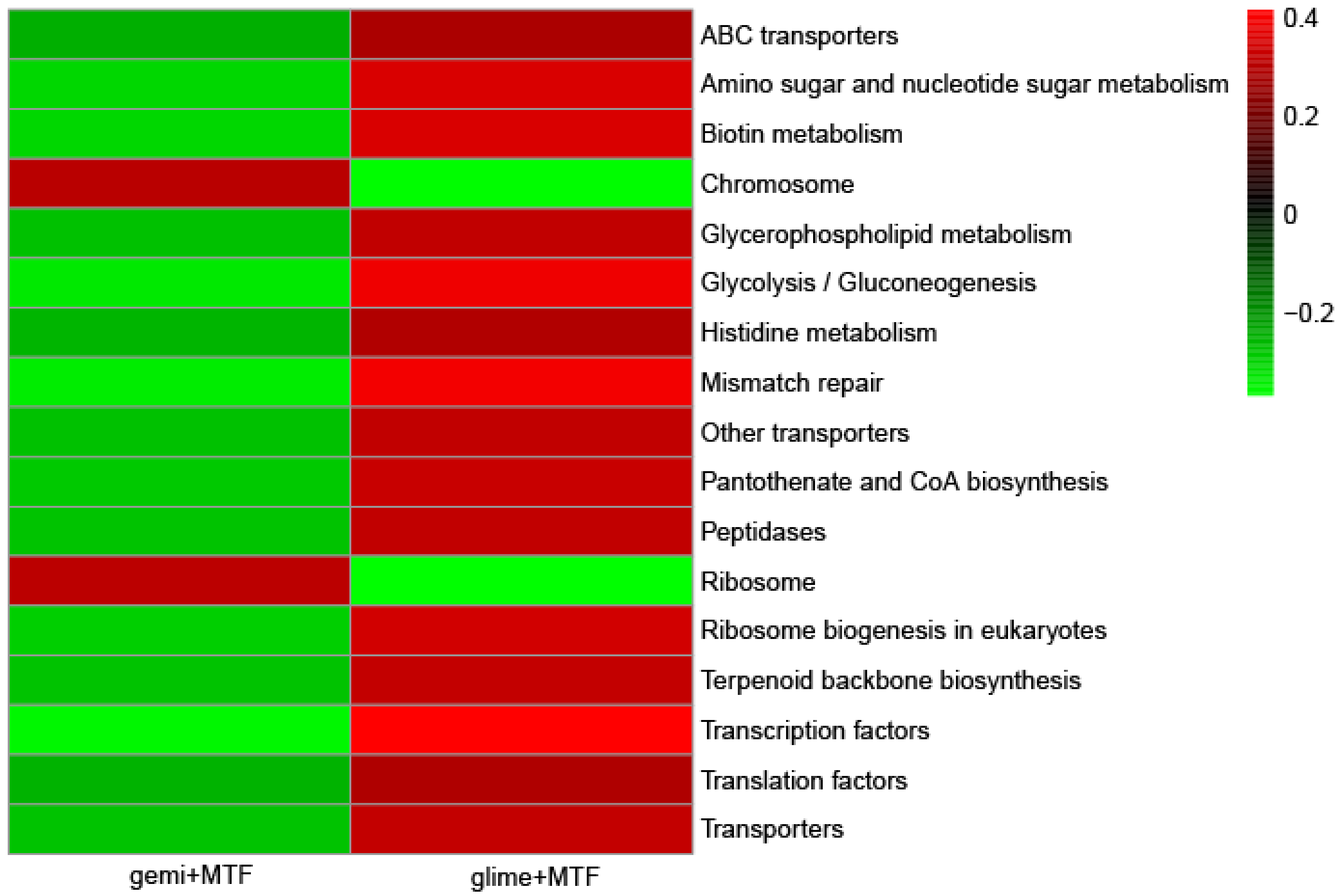

Supplementary Figure S7. Heatmap of the Pearson's correlation analysis between clinical parameters with changes in amino acids. *FDR-adjusted $P < 0.1$. Key: AUCglc, area under curve of oral glucose tolerance test; proins/ins, proinsulin/insulin ratio; HOMA-IR, homeostasis model assessment for insulin resistance; Bwt, body weight; WC, waist circumference; FM, fat mass; VFA, visceral fat area; PAI, plasminogen activator inhibitor; hsCRP, high-sensitivity C-reactive protein; BCAA, branched-chain amino acids; AAA, aromatic amino acids

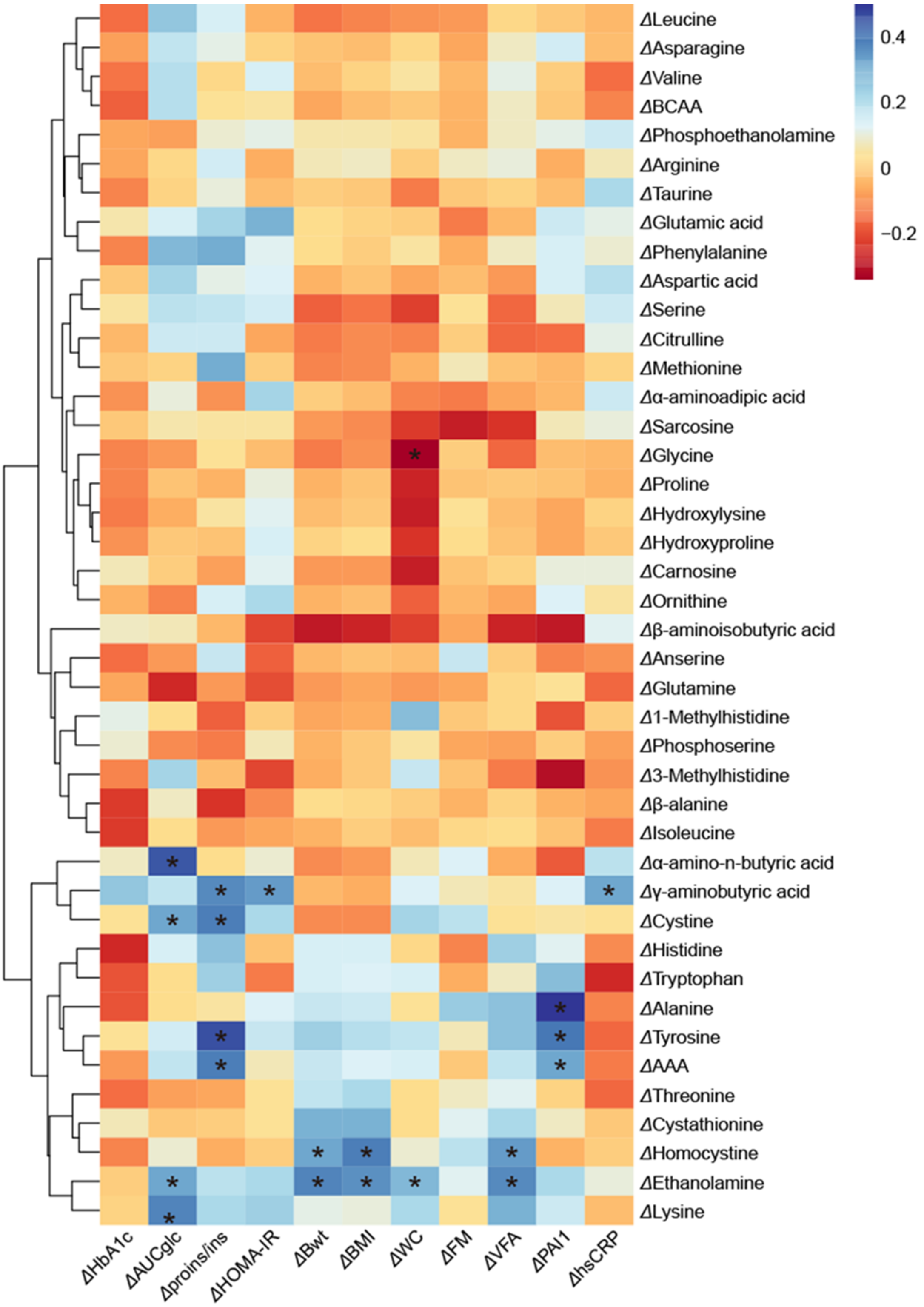

Supplementary Table S1. Changes in amino acids at the baseline and after 24 weeks of treatment with the gemigliptin–metformin or glimepiride–metformin combination

Variables	Gemigliptin + Metformin (*n* = 34)			Glimepiride + Metformin (*n* = 34)			*P* for delta comparison
	Baseline	24 weeks	*P**	Baseline	24 weeks	*P**	
Alanine	377.7 ± 76.7	416.6 ± 71.3	0.018	354.7 ± 61.7	380.2 ± 51.6	0.098	0.417
β-alanine	3.8 ± 0.4	3.7 ± 0.4	0.499	3.9 ± 0.5	3.8 ± 0.3	0.277	0.769
Anserine	0.08 ± 0.10	0.06 ± 0.09	0.653	0.08 ± 0.10	0.08 ± 0.11	0.951	0.682
α-aminoadipic acid	1.3 ± 0.4	1.4 ± 0.5	0.942	1.3 ± 0.4	1.5 ± 0.6	0.392	0.342
α-amino-n-butyric acid	26 ± 6.6	20.7 ± 5.7	<0.001	26.1 ± 7.4	21.1 ± 4.1	0.008	0.863
γ-aminobutyric acid	0.37 ± 0.12	0.35 ± 0.15	0.719	0.34 ± 0.15	0.41 ± 0.2	0.336	0.119
β-aminoisobutyric acid	1.7 ± 1.1	1.8 ± 1.2	0.942	1.7 ± 0.7	1.7 ± 0.9	0.978	0.984
Arginine	76.3 ± 15.4	73.7 ± 15.2	0.438	77.7 ± 15.8	78.4 ± 15.1	0.869	0.333
Asparagine	48.8 ± 10.6	54.3 ± 11.9	<0.001	50 ± 5.7	53.5 ± 7.3	0.027	0.231
Aspartic acid	5.7 ± 1.7	6.8 ± 3.5	0.279	5.3 ± 1.8	6.7 ± 3.7	0.260	0.758
Carnosine	0.11 ± 0.21	0.03 ± 0.05	0.106	0.13 ± 0.18	0.04 ± 0.05	0.029	0.879
Citrulline	29.8 ± 8.5	26.8 ± 8.9	0.008	29.9 ± 6.1	29.2 ± 7.3	0.681	0.110
Cystathionine	0.18 ± 0.28	0.22 ± 0.18	0.738	0.10 ± 0.14	0.29 ± 0.30	<0.001	0.023
Cystine	9.2 ± 6.3	10.8 ± 7.5	0.219	9.6 ± 7.1	9.2 ± 4.8	0.741	0.089
Ethanolamine	7.5 ± 1.5	7.0 ± 1.5	0.102	7.5 ± 1.4	7.8 ± 1.7	0.460	0.022
Glutamic acid	86.4 ± 33.6	82.7 ± 24.6	0.569	80.3 ± 18.9	76.5 ± 21.9	0.432	0.998
Glutamine	514.4 ± 101.6	559.1 ± 88.9	0.018	567.8 ± 68.4	588.2 ± 60.4	0.260	0.178
Glycine	198 ± 31.4	214.5 ± 36.4	0.038	203.3 ± 39.1	237.9 ± 72.4	0.008	0.116
Histidine	76.5 ± 10	82.3 ± 11.4	0.039	78.8 ± 9.0	81.2 ± 9.1	0.432	0.231
Homocysteine	0.06 ± 0.08	0.10 ± 0.11	0.204	0.02 ± 0.04	0.10 ± 0.13	0.014	0.296
Hydroxylysine	0.3 ± 0.22	0.31 ± 0.18	0.942	0.27 ± 0.22	0.82 ± 2.35	0.398	0.199
Hydroxyproline	14.8 ± 14.3	12.1 ± 6.0	0.499	13.5 ± 5.2	28.2 ± 54.7	0.286	0.070
Isoleucine	75.4 ± 12.8	77.4 ± 12.1	0.569	76.3 ± 14.8	74.6 ± 14.9	0.529	0.209
Leucine	141.8 ± 24.7	142.3 ± 20.3	0.942	142.9 ± 26.2	140.1 ± 25.5	0.591	0.527

Lysine	189.5 ± 24.8	189.2 ± 26.7	0.960	187.8 ± 25.2	186.4 ± 24.7	0.841	0.858
Methionine	31.2 ± 10.1	24.7 ± 4.7	<0.001	30.5 ± 9.2	26.1 ± 4.9	0.018	0.304
1-Methylhistidine	2.5 ± 2.9	3.3 ± 3.4	0.438	2.3 ± 2.2	3.5 ± 3.4	0.286	0.763
3-Methylhistidine	3.3 ± 1.1	3.7 ± 1.4	0.167	3.5 ± 1.2	3.7 ± 1.4	0.432	0.487
Ornithine	73.5 ± 20.9	68.6 ± 20.5	0.305	70.2 ± 13.5	70.3 ± 25.0	0.999	0.345
Phenylalanine	64.5 ± 10.7	64.6 ± 11.1	0.942	63.5 ± 8.6	64.7 ± 9.1	0.591	0.633
Phosphoethanolamine	1.5 ± 0.4	1.5 ± 0.3	0.872	1.5 ± 0.3	1.4 ± 0.3	0.771	0.967
Phosphoserine	0.03 ± 0.07	0.02 ± 0.04	0.499	0.02 ± 0.05	0.02 ± 0.03	0.826	0.506
Proline	154.8 ± 41.7	161.2 ± 37.8	0.352	160.8 ± 37.4	174.5 ± 80.9	0.472	0.605
Sarcosine	12.9 ± 6.2	12.3 ± 7.6	0.771	12.9 ± 6.8	11.9 ± 6.2	0.524	0.802
Serine	110.5 ± 13.9	112.1 ± 21.7	0.81	113.1 ± 21.3	120.2 ± 25.8	0.419	0.400
Taurine	42.4 ± 8.4	43.4 ± 8.0	0.637	43.1 ± 8	44.3 ± 5.9	0.436	0.887
Threonine	110.4 ± 18.4	116 ± 22.7	0.167	127.2 ± 32.9	125.6 ± 23.1	0.807	0.145
Tryptophan	46.5 ± 6.7	56.9 ± 9.2	<0.001	44.7 ± 6.2	55.6 ± 11.2	<0.001	0.816
Tyrosine	67.4 ± 12.6	65.2 ± 11.6	0.411	64.2 ± 9.5	67.4 ± 13.4	0.260	0.035
Valine	273.9 ± 41.8	268.8 ± 34.3	0.637	274.3 ± 46.1	269.6 ± 45.3	0.640	0.971
BCAA	490.2 ± 76.4	488.6 ± 62.9	0.942	497 ± 84.2	482.8 ± 83.8	0.432	0.455
AAA	178.3 ± 25.4	186.7 ± 27.4	0.136	172.4 ± 18.9	187.7 ± 28.3	0.008	0.233

*P values were adjusted using the Benjamini–Hochberg method for multiple comparisons.

Key: BCAA, branched-chain amino acids; AAA, aromatic amino acids.
